# Involvement of ABC-transporters and acyltransferase 1 in intracellular cholesterol-mediated autophagy in bovine alveolar macrophages in response to the *Bacillus Calmette-Guerin* (BCG) infection

**DOI:** 10.1186/s12865-020-00356-x

**Published:** 2020-05-12

**Authors:** Jinrui Xu, Yanbing Zhou, Yi Yang, Cuiping Lv, Xiaoming Liu, Yujiong Wang

**Affiliations:** 1 Key Laboratory of Ministry of Education for Conservation and Utilization of Special Biological Resources in the Western, Yinchuan, China; 2 College of Life Science, Ningxia University, Yinchuan, 750021 Ningxia China

**Keywords:** *Mycobacterium tuberculosis*, Bovine, Macrophage, Cholesterol metabolism, Autophagy

## Abstract

**Background:**

Understanding pathogenic mechanisms is imperative for developing novel treatment to the tuberculosis, an important public health burden worldwide. Recent studies demonstrated that host cholesterol levels have implications in the establishment of *Mycobacterium tuberculosis* (*M. tuberculosis*, Mtb) infection in host cells, in which the intracellular cholesterol-mediated ATP-binding cassette transporters (ABC-transporters) and cholesterol acyltransferase1 (ACAT1) exhibited abilities to regulate macrophage autophagy induced by *Mycobacterium bovis* bacillus Calmette–Guérin (BCG).

**Results:**

The results showed that a down-regulated expression of the ABC-transporters and ACAT1 in primary bovine alveolar macrophages (AMs) and murine RAW264.7 cells in response to a BCG infection. The inhibited expression of ABC-transporters and ACAT1 was associated with the reduction of intracellular free cholesterol, which in turn induced autophagy in macrophages upon to the Mycobacterial infection. These results strongly suggest an involvement of ABC-transporters and ACAT1 in intracellular cholesterol-mediated autophagy in AMs in response to BCG infection.

**Conclusion:**

This study thus provides an insight into into a mechanism by which the cholesterol metabolism regulated the autophagy in macrophages in response to mycobacterial infections.

## Background

Tuberculosis (TB) remains a serious infectious disease worldwide, which is primarily caused by the infection of *Mycobacterium tuberculosis* (*M. tuberculosis*, Mtb), a member of the *Mycobacterium tuberculosis* complex (MTBC) [1, 2]. The MTBC is a group of highly related pathogens that are spread via an airborne route and are taken up by alveolar macrophages (AMs) in their respective hosts, of which includes bovine and human strains of the tuberculosis bacillus [3]. In this regard, *Mycobacterium tuberculosis (M. tuberculosis*, *Mtb)*, *Mycobacterium bovis* (*M. bovis*) and the *Mycobacterium bovis* BCG vaccine strain demonstrate distinct virulence, host range and metabolism. Although, the pathogenic roles of above bacilli are expensively studied, the role of metabolic differences in pathogenicity remains poorly understood [4].

Autophagy is an intracellular catabolic process that helps maintain homeostasis or the removal of invading pathogens *via* a lysosomal degradation process [5–8]. In spite of a live attenuated vaccine against tuberculosis caused by *Mtb,* the *Mycobacterium bovis* BCG maintains an ability to induce autophagy responses [9, 10], and evade phagosome maturation and autophagic degradation [11].

A compelling body of evidence has shown that the systemic cholesterol level is associated with the host immunity. Indeed, in addition to atherosclerosis and Alzheimer’s disease, an abnormal cholesterol metabolism has been implicated in several lung diseases, including the development of TB [12]. Cholesterol metabolism is central to *Mtb*’s unusual ability to survive in macrophages and provide insights into potential targets for novel therapeutics [12, 13]. In this regard, the metabolic imbalance caused by an infection of *Mtb* leads the formation of lipid droplets in macrophages, and the accumulation of lipids forms in foam cells, in order to provide a sufficient energy source for the Mycobacteria survival in host cells [14]. Recent studies in immunometabolism demonstrate the intimate link between the metabolic states of immune cells in *Mtb* infections [15], in which the host lipid metabolism is associated with the *Mtb-*induced cell autophagy [16]. We sought to determine whether BCG-induced autophagy was able to be mediated by cholesterol metabolism.

It has been well documented that ATP-binding cassette transporters (ABC-transporters) [17], and cholesterol acyltransferase [18] play crucial roles in cellular cholesterol balance of immune cells such as macrophages and monocytes [19]. In this regard, ABC-transporters are a family of proteins that utilize the ATP-hydrolyzed energy to pump substrates across lipid bilayers [20]. In monocytes/macrophages, ABC-transporters have been demonstrated to involve in reverse cholesterol transport (RCT) and forestall atherosclerotic lesion progression [21]. In this context, the PPARγ-LXRα-ABCA1/ABCG1 signaling is involved in regulating cholesterol efflux in macrophages, where an activation of liver X receptor alpha (LXRα) directly induces the expression of the membrane ATP-binding cassette transporters, such as ABCA1 and ABCG1, which in turn pumps cholesterol out of cells [22]. Similarly, the cholesterol acyltransferases (ACATs) are exclusively intracellular enzymes that produce cholesteryl ester utilizing free cholesterols as the substrates, which is a key procession in maintaining cellular cholesterol homeostasis [23]. To date, two mammal ACATs, the ACAT1 and ACAT2 have been identified [24]. The ACAT1 is an enzyme that resides in the endoplasmic reticulum (ER) membrane of cells. It is also a major isoenzyme in macrophages [25]. An increased number of evidences showed that the ACAT1 was involved in the pathogenesis of many human diseases such as atherosclerosis, Alzheimer’s disease (AD) and cancers, and it has been investigated as a potential target for treatment of these diseases [26].

With regard to bovine AMs, our previous RNA-Seq analysis revealed an alteration of ABC-transporters and ACAT1 in BCG-infected primary bovine AMs as compared to the naïve AMs (Suppl. Fig. S1). Together with aforementioned findings, we therefore hypothesize that the BCG-altered ABC-transporters and ACAT1 may have an important implication in the regulation of intracellular cholesterol level and autophagy in macrophages in response to Mycobacterial infections. Our study will provide useful information to further unveil the function and mechanism of autophagy in mycobacterium-host interactions.

## Results

### Alterations of ABC-transporters and ACAT1 in BCG-infected macrophages

**Fig. 1:**
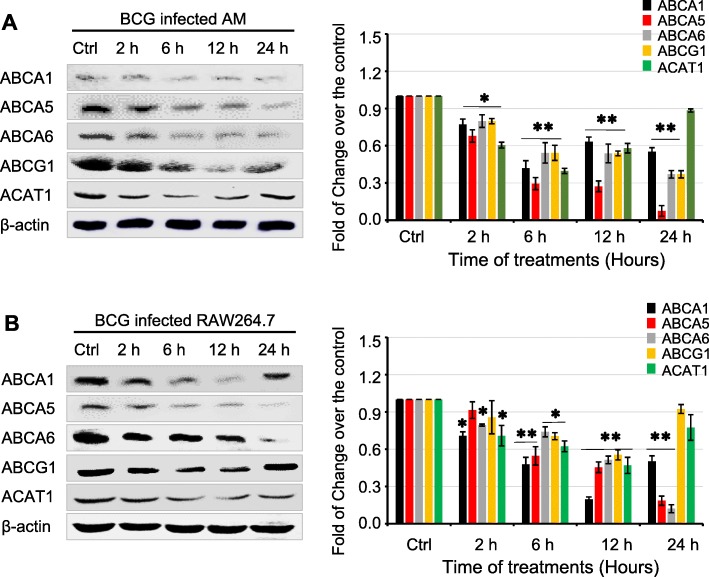
The dynamic changes of the expression of ABC-transporters and ACAT1 in macrophages response to a BCG infection. Primary bovine alveolar macrophages (AMs) and murine macrophage RAW264.7 cells were infection with BCG at a dose of 10 for indicated time periods, abundances of ABC-transporters and ACAT1 were determined by an immunoblotting (IB) assay. **a** Representative images of blots for indicated proteins of interest of BCG-infected primary bovine AMs probed with corresponding antibodies (Left panel), and the relative expression of proteins semi-quantified by an optical densitometry analysis (Right panel) demonstrated a dynamic change of protein expression (**b**) Representative images of blots for indicated proteins of interest of BCG-infected murine macrophage RAW264.7 cells probed with corresponding antibodies (Left panel), and the relative expression of proteins semi-quantified by an optical densitometry analysis (Right panel) demonstrated a dynamic change of protein expression. Both the BCG-infected bovine AMs and RAW264.7 cells exhibited the lowest expression of ABCA1, ACAT1 ABCG1, ABCA5 and ABCA6 at 6, 6, 12, 24 and 24 h post the BCG infection, respectively. Data were expressed as mean ± SEM from three independent experiments. Compared to non-infection controls, *: *p* < 0.05; **: *p* < 0.01

For further understanding the immune response macrophage induced by *Mycobacterium tuberculosis* infection at molecular levels, we analyzed RNA-Seq data in bovine alveolar macrophage (AM) at 12 h post a BCG infection. The sequencing data uncovered 1111 differential expression of mRNA between the infected group and the non-infected group, of which 426 genes were up-regulated, and 685 were down-regulated (Suppl. Fig.S1 and Table 1). Among them, the ABC-transporters *ABCA5*, *ABCA6*, and *ABCA10* genes were down-regulated by more than 1.5 folds in primary bovine AMs infected with BCG (Table 1). Of note, the ABCA5 was reported to correlate with cholesterol efflux in macrophage, while little is known about functions of ABCA6 and ABCA10 [27], suggesting the BCG-altered ABC transporters may have an important implication in the regulation of intracellular cholesterol in macrophages. In order to validate the RNA-Seq findings and explore the changes of other ABC transporters in macrophages, the abundance of transcripts of *ABCG1, ABCA1, ABCA5* and *ABCA6*, as well as the *acyltransferase 1* (*ACAT1*), a gene related to intracellular cholesterol, in both primary bovine AMs and RAW264.7 cells with BCG for 12 h was evaluated by a qRT-PCR assay (Suppl. Fig. S2). As expected, the expression of all above tested genes was strikingly down-regulated in macrophages in response to the BCG infection (Suppl. Fig. S2). In agreement with the finding of transcripts, the down-regulated ABC transporters and ACAT1 was further corroborated by an immunoblotting assay, i.e. less abundant ABCG1, ABCA1, ABCA5, ABCA6 and ACAT1 proteins were observed in both BCG-infected primary bovine AMs (Fig. 1a) and murine RAW264.7 cells (Fig. 1b), in comparison with the naïve macrophages. Of interest, the least ABCA1 and ACAT1 proteins in BCG-infected bovine AMs were observed at post infection 6 h, while the ABCG1 was at 12 h, and the least ABCA5 and ABCA6 were at 24 h post infection (Fig. 1a). Accordingly, the least ABCG1, ABCA1 and ACAT1 proteins in BCG-infected RAW264.7 cells were found at 12 h post infection, while the least ABCA5 and ABCA6 were at 24 h post infection (Fig. 1b).

**Table 1:** The differentiate expression of ABC transports, ABCA6, ABCA5 and ABCA10 genes in primary bovine alveolar macrophages in response to BCG infection

Gene	BCG12_FPKM	C12_FPKM	log2 (fold change)	*P* value	*Q* value
*ABCA6*	1.94886	5.71408	−1.55189	0.001982	0.025436
*ABCA10*	0.254202	3.55728	−3.80673	7.05E-12	1.49E-09
*ABCA5*	2.28543	7.0428	−1.62368	0.001165	0.016954

### The BCG infection increases intracellular cholesterol in macrophages

**Fig. 2:**
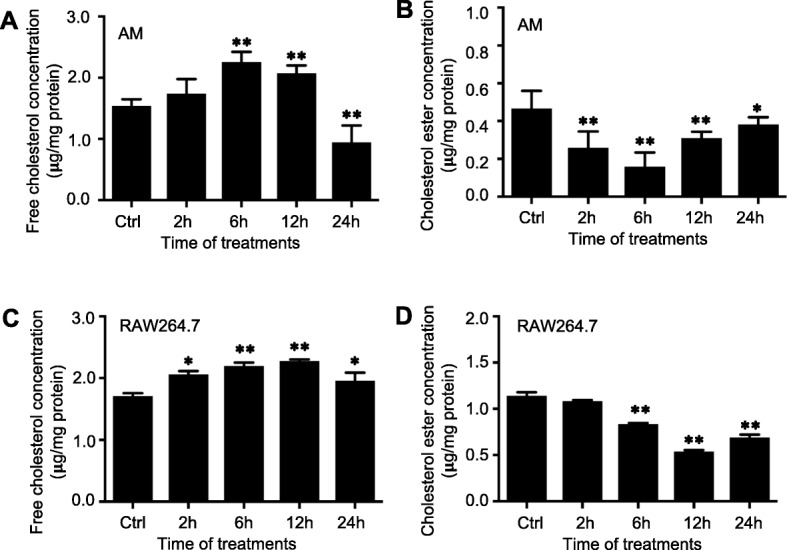
The BCG infection altered intracellular levels of cholesterol and cholesterol ester in macrophages. Bovine AMs and murine macrophage RAW264.7 cells were infected with BCG at a dose of 10 for indicated times before their intracellular levels of cholesterol and cholesterol ester were ascertained. **a**-**b** Intracellular levels of cholesterol (**a**) and cholesterol ester (**b**) of primary bovine AMs altered by the infection of BCG. **a** The content of intracellular free cholesterol was increased gradually and reached its peak at 6 h post infection before it significantly decreased from 12 h afterward; **b** while the intracellular cholesterol ester content in AMs was significantly decreased and reached the lowest level at 6 h and then dramatically increased at 12 and afterward. **c**-**d** Intracellular levels of cholesterol (**c**) and cholesterol ester (**d**) of murine RAW264.7 cells altered by BCG. **c** The intracellular free cholesterol was increased and reached its peak at 12 h but reduced at 24 h post infection. A significant change of intracellular cholesterol was observed between 2 h, 6 h and 12 h after infection as compared with the control group; in contrast, **d** the intracellular cholesterol ester was significantly decreased and reached the lowest level at 12 h but increased at 24 h post infection. A significant change of intracellular cholesterol ester was determined between cells at 6 h, 12 h and 24 h following the infection, as compared with the control group. Data were expressed as mean ± SEM from three independent experiments. Compared to non-infection control, *: *p* < 0.05, **: *p* < 0.01

The ABC-transporters ABCG1, ABCA1, ABCA5 participate in the reverse cholesterol transport from macrophages and ACAT1 converts free cholesterol to cholesteryl esters [28]. A down regulation of these proteins may imply an increase of intracellular cholesterol. Indeed, a dynamic change of intracellular cholesterol and cholesterol ester with time was observed in both bovine AMs and macrophages in response to the BCG infection (Fig. 2). In this context, intracellular levels of free cholesterol (Fig. 2a) and cholesterol ester (Fig. 2b) of primary bovine AMs were gradually increased and decreased, respectively. The intracellular free cholesterol (Fig. 2a) and cholesterol ester (Fig. 2b) reached their peaks at 6 h post infection before they respectively decreased and increased from 12 h afterward. Consistently, the dynamic changes of intracellular free cholesterol (Fig. 2c) and cholesterol ester (Fig. 2d) were also detected in BCG-infected RAW264.7 cells. The intracellular free cholesterol was increased and reached its peak at 12 h but reduced at 24 h post infection (Fig. 2c); while the intracellular cholesterol ester was significantly decreased and reached the lowest level at 12 h but increased at 24 h post infection (Fig. 2d). These results may indicate that the increased intracellular cholesterol is a consequence of BCG-inhibited expression of ABC-transporters and ACAT1.

### Infection of BCG induces autophagy in macrophages

**Fig. 3:**
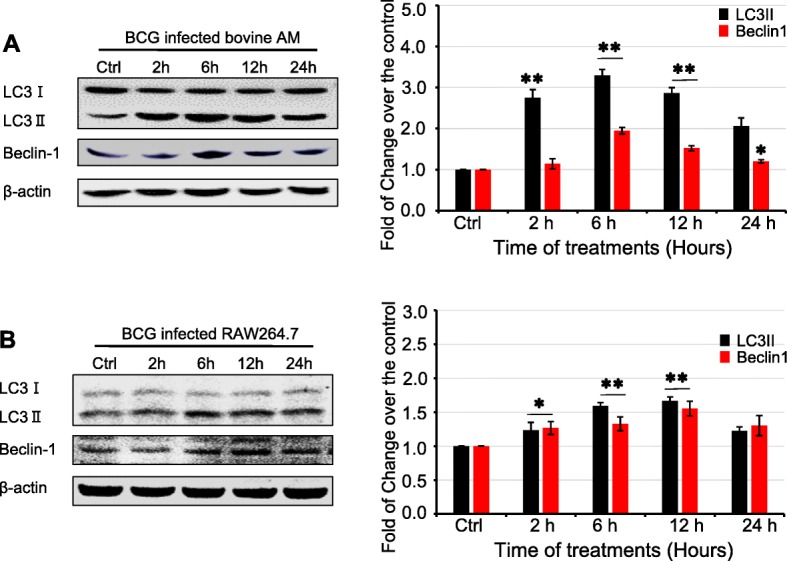
BCG infection induced the expression of autophagy-related proteins in macrophages. Bovine AMs and murine macrophage RAW264.7 cells were infected with BCG at a dose of 10 for indicated times, and the expression of autophagy-related proteins LC3I/II and Beclin1 was determined by an immunoblotting (IB) assay. **a** Representative images of blots for LC3I/II and Beclin1 in BCG-infected primary bovine AMs (left panel), and the relative expression of proteins semi-quantified by a densitometry analysis (right panel) demonstrated an increased expression of autophagy-related proteins. **b** Representative images of blots of LC3I/II and Beclin1 in BCG-infected murine macrophage RAW264.7 cells (left panel), and their relative expression semi-quantified by a densitometry analysis (right panel) indicated an induced autophagy by BCG infection. Data were expressed as mean ± SEM from three independent experiments. Compared to non-infection controls, *: *p* < 0.05; **: *p* < 0.01

Accumulating evidences have demonstrated that autophagy can be induced by cholesterol in Mycobacteria-infected macrophages [29]. In line with other studies, a significant increased expression of autophagy markers LC3II/I and Beclin1 was observed in primary bovine AMs (Fig. 3a) and RAW264.7 cells (Fig. 3b). Interestingly, the BCG-induced expression of autophagy markers showed a comparably dynamic trend with the change of intracellular cholesterol in both cell types (Fig. 2), indicating that the intracellular cholesterol may play a role in regulating autophagy in BCG-infected macrophages, which needs further investigation.

### The involvement of ACAT1-mediated intracellular cholesterol in autophagy of macrophages in response to BCG infection

**Fig. 4:**
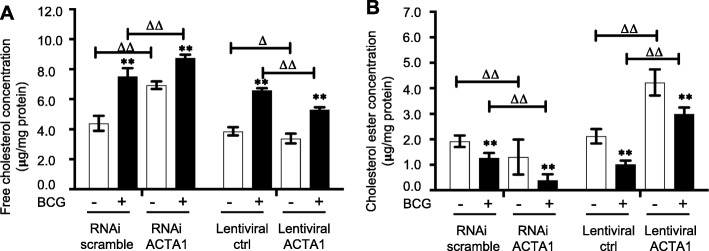
An ACAT1-mediated alteration of intracelluar cholesterol and cholesterol ester in macrophages in response to BCG infection. Murine RAW264.7 cell lines overexpressing and silencing ACAT1 were generated by lentiviral infections with vectors overexpressing ACAT1 or shRNA to ACAT1, respectively. Control RAW264.7 cell lines infected with appropriate control lentiviral vectors were also generated. The transgenic RAW264.7 cells were infected with BCG at a dose of 10 for 12 h prior to being harvested for assessment of intracellular cholesterol (**a**) and cholesterol ester (**b**). **a** An overexpression of ACAT1 exhibited an ability to significantly suppress BCG-induced intracellular cholesterol, while knockdown of ACAT1 showed an enhanced level of intracellular cholesterol in RAW264.7 cells. **b** An overexpression of ACAT1 significantly restored BCG-repressed intracellular cholesterol ester, while knockdown of ACAT1 further reduced the BCG-repressed cholesterol ester in RAW264.7 cells. Data were expressed as mean ± SEM from three independent experiments. Compared to non-BCG-infected cells, *: *p* < 0.05; **: *p* < 0.01; compared to non-control lentivirus-infected cells, ^∆^: *p* < 0.05; ^∆∆^: *p* < 0.01

**Fig. 5:**
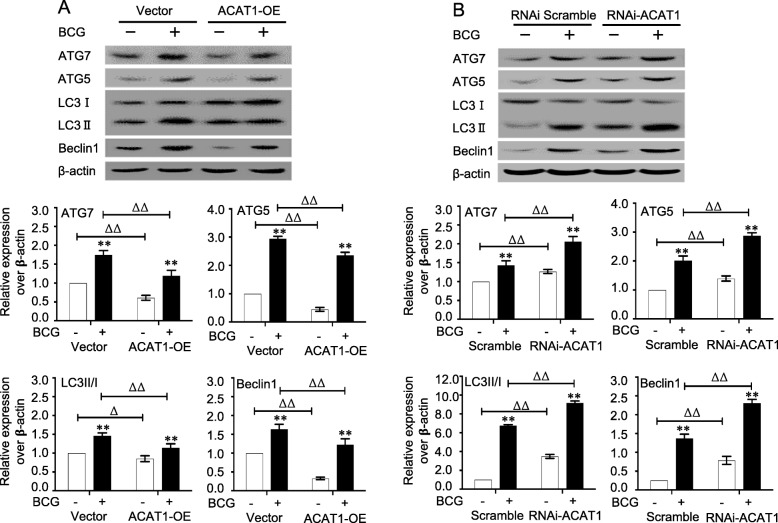
The impact of ACAT1 on the BCG-induced autophagy in RAW264.7 cells. The ACAT1-overexpressed and silenced RAW264.7 cells were infected with BCG at a dose of 10 for 12 h before the pathogen-induced autophagy was determined by accessing autophagy-related proteins. **a** Representative blots showed an inhibition of BCG-induced autophagy-related proteins ATG7, ATG5, LC3II/I and Beclin1 in RAW264.7 cells overexpressing ACAT1 (top panel), and the relative expression semi-quantified by a densitometry analysis (bottom panel) demonstrated an significant reduction of autophagy-related proteins in comparison with control cells. **b** Representative blots displayed an increase of BCG-induced autophagy-related proteins ATG7, ATG5, LC3II/I and Beclin1 in RAW264.7 cells with a silence of ACAT1 (top panel), and the relative expression semi-quantified by a densitometry analysis (bottom panel) demonstrated an significant increase of autophagy-related proteins in comparison with uninfected control cells. Data were expressed as mean ± SEM from three independent experiments. Compared to non-BCG-infected cells, *: *p* < 0.05; **: *p* < 0.01; compared to non-control lentivirus-infected cells, ^∆^: p < 0.05; ^∆∆^: p < 0.01

**Fig. 6:**
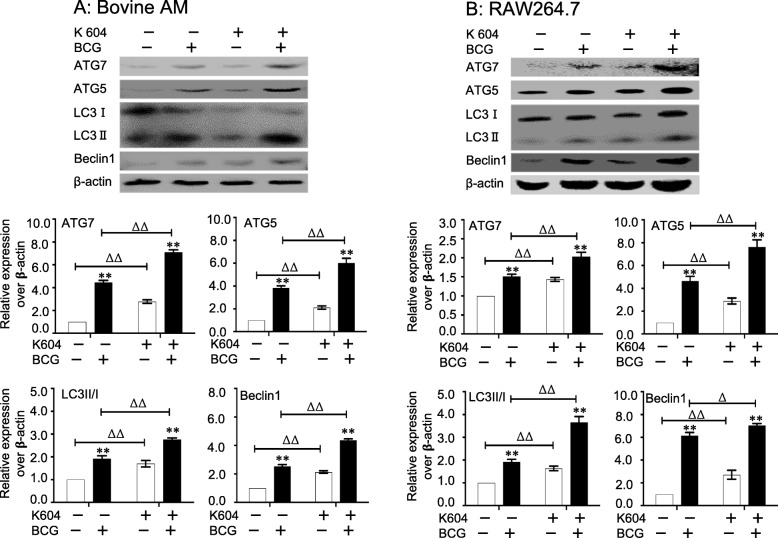
The impact of ACAT1 signaling on the BCG-induced autophagy in RAW264.7 cells. The primary bovine AMs and RAW264.7 cells were infected with BCG at a dose of 10 in the presence of ACAT1 signaling inhibitor K604 for 12 h before the autophagy was determined by accessing autophagy-related proteins. **a** Representative blots demonstrated an increased expression of BCG-induced autophagy-related proteins ATG7, ATG5, LC3 and Beclin1 in primary bovine AMs in the presence of K604 (top panel), and the relative expression semi-quantified by a densitometry analysis (bottom panel) demonstrated an significant increase of autophagy-related proteins in comparison with cells without K604. **b** Representative blots demonstrated an increased expression of BCG-induced autophagy-related proteins ATG7, ATG5 LC3II/I and Beclin1 in RAW264.7 cells in the presence of K604 (top panel), and the relative expression semi-quantified by a densitometry analysis (bottom panel) demonstrated an significant increase of autophagy-related proteins in comparison with cells without K604. Data were expressed as mean ± SEM from three independent experiments. Compared to non-BCG-infected cells, *: *p* < 0.05; **: *p* < 0.01; compared to cells in the absence of K604, ^∆^: *p* < 0.05; ^∆∆^: *p* < 0.01

*The ACAT1* gene has been demonstrated to correlate with intracellular cholesterol and autophagy [26]. In order to further validate the involvement of the alteration of ACAT1 in BCG-infected macrophages, RAW264.7 stable cell lines overexpressing and silencing ACAT1 were generated by lentiviral vector-mediated gene transduction (data not shown). As expected, the overexpression of ACAT1 significantly decreased the BCG-induced intracellular free cholesterol, while the silence of ACAT1 expression led an increased BCG-induced intracellular free cholesterol (Fig. 4a). In consistence, an overexpression of ACAT1 restored the BCG-inhibited intracellular cholesterol ester, while a silence of ACAT1 aggravated the suppression of BCG-reduced intracellular cholesterol ester (Fig. 4b). Of importance, the ACAT1-altered intracellular cholesterol and cholesterol ester were correlated with the abundance of protein markers of autophagy in macrophages infected with BCG (Fig. 5). An overexpression ACAT1 reduced the BCG-induced expression of autophagy-related proteins ATG5, ATG7 LC3II/I and Beclin1 (Fig. 5a), while knocking-down of ACAT1 expression enhanced the BCG-induced autophagy proteins in RAW264.7 cells (Fig. 5b). This funding was further corroborated by an experiment using ACAT1 specific inhibitor K604 (Fig. 6). In the presence of K604, the BCG-induced autophagy-related proteins ATG5, ATG7, LC3II/I and Beclin1 were significantly increased in both primary bovine AMs (Fig. 6a) and murine RAW264.7 cells (Fig. 6b), as compared with their respective controls in the absence of K604. These results strongly suggest an involvement of ACAT1 in regulation of intracellular cholesterol and autophagy of macrophages in response to BCG Infection.

## Discussion

Both of bovine and human strains of the tuberculosis bacillus, *Mtb* and *Bovis tuberculosis* are members of MTBC. Despite they demonstrate distinct difference in virulence, host range and metabolism, the metabolic differences of these bacilli in pathogenicity remains exclusive [4]. The ability of the pathogen *Mtb* to metabolize steroids like cholesterol plays a key role in the virulence and pathogenesis [30]. Cholesterol, a major structural component of animal cell membranes, is thought to be involved in immune regulations [31] and the development of *Mtb* infection [30]. In this regard, *Mtb* is able to import and metabolize host cholesterol during infection, which is critical for the maintenance of *Mtb* infection [30]. In addition, cholesterol also is a critical carbon source during latent *Mtb* infection, during which *Mtb* is capable of using cholesterol as a carbon source [32]. Moreover, the cholesterol of macrophage host has been shown to facilitate the entry of mycobacteria into macrophages [33], where mycobacteria-activated macrophages effectively transfer the phagocytosed pathogens to the destructive microenvironment of lysosomes [34]. As an important component of mammalian cells with many basic cellular functions, the intracellular cholesterol level is regulated many factors such as Acyl-CoA: cholesterol acyltransferase 1 (ACAT1) and ABC transporters [35]. Functionally, the ACAT1 is the major isoenzyme in macrophages [25], which converts cholesterol to cholesterol esters, and plays important roles in lipoprotein assembly, dietary cholesterol absorption, and intracellular cholesterol metabolism [36].

In this study, a down-regulated ATP-banding transporters family members related to cholesterol efflux, were observed in BCG-infected bovine AMs by RNA-seq analysis. The results suggested that BCG infection-altered the intracellular cholesterol was regulated by ABC transporter-mediated cholesterol efflux. This hypothesise was supported by alterations of the expression of ABCA5, ABCA6, ABCA1, ABCG1 at both transcriptional and translational levels in AMs and murine RAW264.7 cells. On the other hand, the expression of Acyl-CoA: cholesterol acyltransferase1 (ACAT1) was also altered in these cells, suggesting its important role in regulating the intracellular cholesterol in macrophages in response to mycobacterial infections.

Programmed cell death and autophagy are fundamental processes of cell biology intimately involved in the interaction between *Mycobacteria* and their infected phagocytes [37]. In this regard, autophagy can target *Mtb* and promote phagosomal maturation to inhibit the replication of intracellular bacilli. However, the bacilli have an ability to escape from lysosome and survive within their host macrophages by utilizing host resources as early niches for their replication and growth in cells. Emerging evidence suggests that the cholesterol metabolism plays a crucial role in the regulation of macrophage autophagy [31, 38]. In line with these findings, we also observed a trend of that the change of cholesterol in macrophages was consistent with the expression of autophagy-related proteins during BCG infection. Functionally, the ACAT1 is the major isoenzyme in macrophages [24], which plays important roles in lipoprotein assembly, dietary cholesterol absorption, and intracellular cholesterol metabolism [31]. This notion was further corroborated in AMs and RAW264.7 cells by targeting ACAT1 by altering its expression and/or introducing small molecule inhibitor K604.

Indeed, an overexpression or silence of ACAT1 in murine RAW264.7 cells led a respectively decreased and increased intracellular ACAT1 intracellular free cholesterol, but an opposite effect in intracellular cholesterol ester in response to BCG infections. Interestingly, the ACAT1-mediated alterations of intracellular cholesterol were positively correlated with the BCG-induced autophagy in macrophages. Of note, the ACAT1 inhibitor K604 exhibited a similar effect to shRNA-mediated ACAT1 knockdown in RAW264.7 cells, suggesting an involvement of ACAT1-regulated intracellular cholesterol in BCG-induced autophagy. In addition to the ACAT1, ABC transporters, ABCA1 and ABCG1 showed an ability to pump cholesterol out of macrophages [22]. In addition, we also found that ABC-transporters were involved in the alteration of intracellular cholesterol in in both bovine alveolar macrophages and murine RAW264.7 macrophages infected with BCG. These data thus suggest a regulatory role of efflux transporters of macrophages in maintaining intracellular cholesterol and cell autophagy during BCG infection.

## Conclusions

In summary, in the present study, we explored the regulatory roles of ABC-transporters and ACAT1 in cholesterol metabolism of bovine macrophages in response to an infection of *Mycobacterium bovis* vaccine stain BCG. Our results demonstrated that the infection of BCG inhibited the expression of ABC-transporters and ACAT1 in primary bovine AMs and murine RAW264.7 cells, which in turn reduced the intracellular free cholesterol and increased cholesterol ester. Importantly, the BCG-inhibited expression of ABC-transporters and ACAT1, and its consequently reduced intracellular cholesterol was correlated with the BCG-induced autophagy in macrophages, clearly indicating an involvement of ABC-transporters and ACAT1 in intracellular cholesterol-mediated autophagy in macrophages in response to Mycobacterial infections. This study thus highlights an importance of metabolism of intracellular cholesterol in macrophages, aids our better understanding in the innate immune mechanisms during mycobacterial infection and eventually provides useful information for development of host-directed therapy (HDT) strategies in TB treatments.

## Methods

### Primary bovine alveolar macrophages and RAW264.7 cells

This study was approved by the ethics committee for use and care of animals at Ningxia University (Yinchuan, China). Primary bovine AMs were obtained from lungs of one- to two-year-old Simendal cattles bred in a tuberculosis-free herd. The entire lung was sterilely removed post-mortem with a portion of trachea, and was intratracheally infused with 500 mL of D-Hank’s solution (Biotopped, Beijing, China) containing 50 μg/mL of gentamicin, 2.5 μg/mL of amphotericin and 100 μg/mL of penicillin and streptomycin (Hyclone, Logan, USA). The recovered bronchoalveolar lavage fluid (BALF) was filtered by passing a 70 μm-pore nylon cell strainer prior to being collected into sterile beakers and centrifuged at 1000 rpm for 10 min. The cell pellet was then resuspended in 20 mL RPMI-1640 medium (HyClone, Logan, USA) supplemented with 10% Fetal Bovine Serum (FBS, Gibco, Carlsbad, USA), 100 U/mL of penicillin and 100 U/mL of streptomycin. 3 mL of red blood cell (RBC) lysate solution was then added into the cell suspension and gently mixed well for 3 min at room temperature (RT) before the cells were recollected by centrifugation at 1000 rpm for 10 min. The cells were resuspended with RPMI-1640 medium with 10% FBS and counted. The resultant primary cells were seeded at a density of 5 × 10^7^ per tissue culture dish with diameter of 140 mm and cultured in RPMI-1640 medium with 10% FBS for 6 h to 8 h. The unattached cells were removed by rinsing the culture with pre-warmed PBS, and the attached monolayer of primary AMs were dissociated with Tryple™ Express (Thermo Fisher Scientific, Shanghai, China) and harvested by centrifugation. The isolated cells were then re-plated in 6-well plates at a density of 5 × 10^6^/well in RPMI-1640 medium with 10% FBS and cultured for 16–18 h for subsequent experiments. The murine macrophage cell line RAW264.7 was purchased from Shanghai Academy of Life Sciences, Chinese Academy of Sciences (Shanghai, China). Cells were maintained in DMEM medium supplemented with 10% calf serum and penicillin/streptomycin. All cells were cultured in humidified incubators with 5% CO_2_ atmosphere at 37 °C.

### BCG culture and infection

The *M. bovis BCG* vaccine strain was purchased from Chengdu Institute of Biological products (Chengdu, China). BCG bacterial cells were grown in the Middlebrook 7H9 medium (BD Difco, San Jose, CA, USA) supplemented with 10% albumin-dextrose-catalase (ADC) enrichment medium (BD Difco, San Jose, CA, USA) and 0.05% Tween 80 (Sigma, St. Louis, MO, USA) at 37 °C with slow shaking for 2 weeks. The bacteria cells were harvested by centrifugation and re-suspended in the culture medium. The bacterial cell number was titrated by spectrophotometer at wavelength of 600 nm based on an OD600nm of 1.0 equivalent to 1 × 10^8^ mycobacterial cells [39]. The bacteria stocks were aliquot and stored at − 80 °C freezer for subsequent uses. For infection, the macrophage cells were cultured with 6-well plates for 6-8 h before they were infected with BCG at a multiplicity of infection (MOI) of 10 bacteria and then incubated for additionally various times.

### RNA-seq analysis

The sequencing of transcriptome was performed by Beijing Novogene Bioinformatics Technology Company. The sequencing platform was Illumina Hi SeqTM4000. TopHat2 was used to conduct comparative analysis of the reference genome of filtered reads [40]. Each sample of the species was quantitatively analyzed using HTSeq software. SNP Calling and InDel Calling were respectively conducted by the mutation detection software GATK2 [41]. mRNA, lncRNA and TUCP transcript were for quantitatively analyzed using Cuffdiff (http://cole-trapnell-lab.github.io/cufflinks/cuffdiff/index.html) software. Differential expression was carried out using Cuffdiff v2.1.1. Functional classification using the GOSeq Release 2.12 with gene ontology (GO) were carried out on genes that were found to be differentially expressed with an adjusted *p*-value of < 0.05 [41].

### Quantitative RT-PCR

The total RNA of cells was isolated using Trizol reagent per manufacturer’s instruction (Invitrogen, Grand Island, NY, USA). The reverse transcription of first-strand cDNA synthesis was generated using M-MLV reverse transcriptase (TaKaRa, Dalian, China). The quantitative reverse transcription PCR (qRT-PCR) was performed in the Roche Lightcycler 2.0 using TaKaRa SYBR Green I kit (Takara, Dalian, China). The primer sets used for RT-PCR were designed and synthesized in Shanghai Sangon Biotech Inc. (Shanghai, China) by bioinformatics tools using available mRNA sequences (Suppl. Table 1). The relative expression of genes of interest was calculated by accessing the efficiencies and the crossing point deviation of a given gene vs housekeeping β-actin gene. In each independent experiment, The relative gene expression was represented by the fold of change over its respective uninfected control cultures by a 2^-ΔΔCT^ method.

### Immunoblotting analysis

Whole cell extracts were prepared by lysing cell cultures in lysis buffer (50 mM Tris-HCl, pH 7.5, 5 mM EDTA, 150 mM NaCl, 0.5% NP-40) for 60 min on ice. The concentration of soluble protein was determined with Bio-Rad Protein Assay based on the method of Bradford (Bio-Rad Laboratories, Richmond, CA, USA). The clarified lysates (100 μg) were resolved in 8% or 10% sodium dodecyl sulfate (SDS)-polyacrylamide gel (SDS-PAGE) and then transferred to nitrocellulose membranes for immunoblotting assay probed with antibodies to proteins of interest. The primary antibodies used in this study were listed in Suppl. Table 2. All these primary antibodies were applied in a dilution of 1:500–1000. Following extensive washing, protein of interest was detected or visualized with an appropriate HRP-labelled or fluorescence-labelled IRDye (Li-Cor Biosciences, Lincoln, NE, USA) secondary antibody. The blots were then developed using the enhanced Western Bright ECL reagent (Advansta, Menlo Park, CA, United States) or Li-Cor Odassay Scanner (Li-Cor Biosciences). The relative expression of protein was semi-quantified by optical densitometry using ImageJ Software version 1.46 (http://rsb.info.nih.gov/ij/). The densitometric arbitrary unit (A.U.) was used for determined the ratio between the net intensity by calculating values of each sample divided by the β-actin internal control.

### Determination of cholesterol and cholesterol ester

The contents of intracellular cholesterol and cholesterol ester were determined by commercially available cholesterol quantitative assay kit according to manufacturer’s instructions (Sigma, St. Louis, MO, USA). The absorbance was then read at 570 nm. Finally, the contents of intracellular total cholesterol and free cholesterol were calculated comparing to its standard curve, and the amount of cholesterol ester was calculated by the substrate of the content of total cholesterol to the content of free cholesterol.

### Generation of RAW264.7 cell lines overexpressing or silencing ACAT1

To generate a lentiviral vector overexpressing ACAT1, cDNA of murine *acat1* gene (NM_144784) was cloned downstream of the CMV promoter of GV492 lentiviral proviral backbone plasmid (Genechem Co., Ltd., Shanghai, China); to generate lentiviral vectors knocking down endogenous ACAT1 expression, shRNAs targeting sequences of 5’TCGGTCTGGCTAGTATTTG3’, 5’CGTACCTAAGGTTCTTAAA3’ and 5’TAACTGATGTCTACAATAA3’ of murine *acat1* gene (NM_144784) were respectively cloned downstream of the U6 promoter of GV493 lentiviral proviral backbone plasmid (Genechem Co.,Ltd., Shanghai, China). The constructed proviral plasmids were used for generation of respective VSV-G pseudotyped lentiviral vectors Lenti-ACAT1 and Lenti-shRNA-ACAT1 as described elsewhere. To generate murine macrophage RAW264.7 cells overexpressing and silencing ACAT1, RAW264.7 cells were infected with Lenti-ACAT1 and Lenti-shRNA-ACAT1, respectively. The virally transduced cells were then cultured for 72 h following the infection before they were refreshed with a selective medium containing purinomycin for additional 4–5 days. The cell pools with were then used for further experiments after functional determination.

### Suppression of ACAT1 using inhibitor K604

RAW264.7 cells were seeded in a 6-well plate culture dish at a density of 2 × 10^6^/well in DMEM-10% FBS culture medium containing 10 μM of ACAT1 protein inhibitor K604 (MedChemExpress, USA) and BCG at a multiplicity of infection of 10:1. The cells were cultured for 12 h before they were used for analysis.

### Statistical analysis

All data collected in this study were from at least three independent biological repeated experiments, which were analysed using SPSS statistics 22.0 (SPSS Inc., Chicago, IL, USA). The data were presented as the mean ± standard derivation (SD). Statistical differences between groups were analysed by one-way analysis of variance (ANOVA), followed by post-hoc Tu-key’s test. The data were presented as the mean ± SD. *P* values < 0.05 were considered as statistically significant.

## Supplementary information

**Additional file 1: **Supplemental file 1 **Figure S1.** The difference of expression primary bovine alveolar macrophages (AMs) in response to of BCG infections. The volcano plots showed the difference in the expression of genes in primary bovine alveolar macrophage (AM) infected with BCG at 12 h at a dose of 10 were analyzed RNA-Seq. The results revealed that there were 1111 differentially expressed genes between the infected group and the non-infected group, of which 426 were up-regulated, and 685 were down-regulated. **Figure S2.** The BCG infection suppresses the expression of ABC-transporters and ACAT1 in bovine alveolar macrophages (AMs). Bovine AMs were infected with BCG at MOI of 10 for 12 h, and the transcripts of ABC-transporters and ACAT1 was assessed by a qRT-PCR assay. (A-E) Inductions of indicated transcripts of bovine alveolar macrophages (AMs) infected with BCG. (A) Fold of changes of ABCA1 transcript over the non-infected cells; (B) Fold of changes of ABCA5 transcript over the non-infected cells; (C) Fold of changes of ABCA6 transcript over the non-infected cells; (D) Fold of changes of ABCG1 transcript over the non-infected cells; (E) Fold of changes of ACAT1 transcript over the non-infected cells. Data represent the mean ± the standard error of the mean (SEM) from three independent experiments. Compared to non-infection control, *: *p* < 0.05, **: *p* < 0.01. Suppl. Table 1. Primer sets of qRT-PCR used in this study.

## Data Availability

The datasets used and/or analysed during the current study are available from the corresponding author on reasonable request.

## Abbreviations

ABCA1: ATP binding cassette subfamily A member 1
ABCA5: ATP binding cassette subfamily A member 5
ABCA6: ATP binding cassette subfamily A member 6
ABCA10: ATP binding cassette subfamily A member 10
ABCG1: ATP-binding cassette subfamily G member 1
ABC-transporters: ATP-binding cassette transporters
ACAT: Acyl coenzyme A:cholesterol acyltransferase
ACAT1: Acyl coenzyme A:cholesterol acyltransferase 1
ACAT2: Acyl coenzyme A:cholesterol acyltransferase 2
AD: Alzheimer’s disease
AM: Alveolar macrophages
ATG5: Autophagy-Related Protein 5
ATG7: Autophagy-Related Protein 7
BCG: Bacillus Calmette-Guerin
DC: Dendritic cells
ER: Endoplasmic reticulum
HDT: Host-directed therapy
LC3: Microtubule associated protein light chain 3
LXRα: Liver X receptor alpha
*M. bovis*: Mycobacterium bovis
MOI: Multiplicity of infection
Mtb: *Mycobacterium tuberculosis*
qRT-PCR: The quantitative reverse transcription PCR
RCT: Reverse cholesterol transport
TB: Tuberculosis
