# Supplementary material for: Involvement of ABC-transporters and acyltransferase 1 in intracellular cholesterol-mediated autophagy in bovine alveolar macrophages in response to the Bacillus Calmette-Guerin (BCG) infection
Source: BMC Immunol. 2020 May 12;21:26. doi: 10.1186/s12865-020-00356-x (PMC7216371; doi:10.1186/s12865-020-00356-x)
Supplement: Supplementary file 1 — Additional file 1: Supplemental file 1 Figure S1. The difference of expression primary bovine alveolar macrophages (AMs) in response to of BCG infections. The volcano plots showed the difference in the expression of genes in primary bovine alveolar macrophage (AM) infected with BCG at 12 h at a dose of 10 were analyzed RNA-Seq. The results revealed that there were 1111 differentially expressed genes between the infected group and the non-infected group, of which 426 were up-regulated, and 685 were down-regulated. Figure S2. The BCG infection suppresses the expression of ABC-transporters and ACAT1 in bovine alveolar macrophages (AMs). Bovine AMs were infected with BCG at MOI of 10 for 12 h, and the transcripts of ABC-transporters and ACAT1 was assessed by a qRT-PCR assay. (A-E) Inductions of indicated transcripts of bovine alveolar macrophages (AMs) infected with BCG. (A) Fold of changes of ABCA1 transcript over the non-infected cells; (B) Fold of changes of ABCA5 transcript over the non-infected cells; (C) Fold of changes of ABCA6 transcript over the non-infected cells; (D) Fold of changes of ABCG1 transcript over the non-infected cells; (E) Fold of changes of ACAT1 transcript over the non-infected cells. Data represent the mean ± the standard error of the mean (SEM) from three independent experiments. Compared to non-infection control, *: p < 0.05, **: p < 0.01. Suppl. Table 1. Primer sets of qRT-PCR used in this study. [file 12865_2020_356_MOESM1_ESM.docx]

**Supplemental files**

**
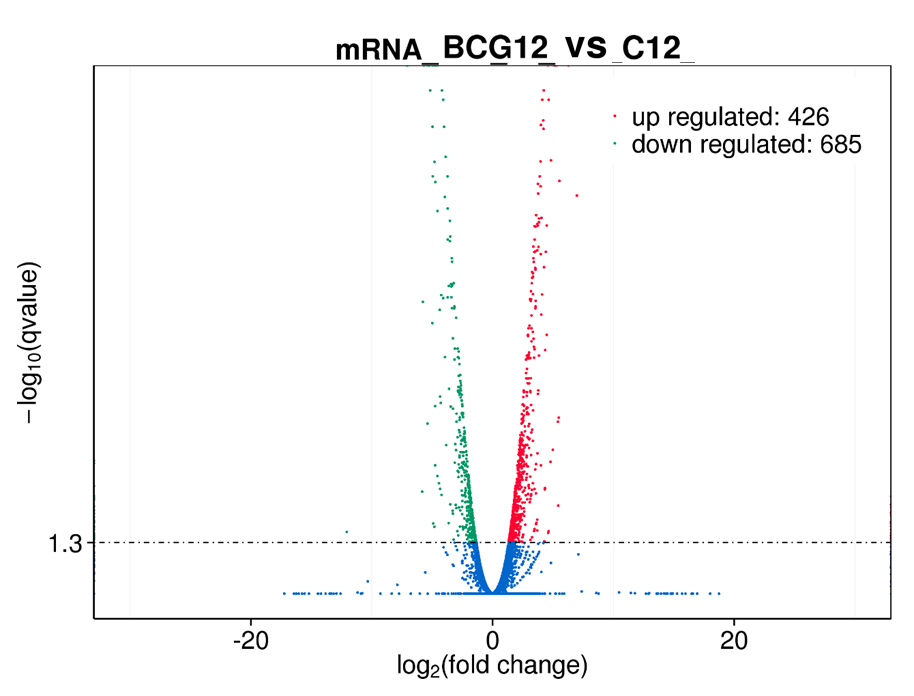
**

**Suppl. Figure S1. The difference of expression primary bovine alveolar macrophages (AMs) in response to of BCG infections.** The volcano plots showed the difference in the expression of genes in primary bovine alveolar macrophage (AM) infected with BCG at 12h at a dose of 10 were analyzed RNA-Seq. The results revealed that there were 1111 differentially expressed genes between the infected group and the non-infected group, of which 426 were up-regulated, and 685 were down-regulated.

**
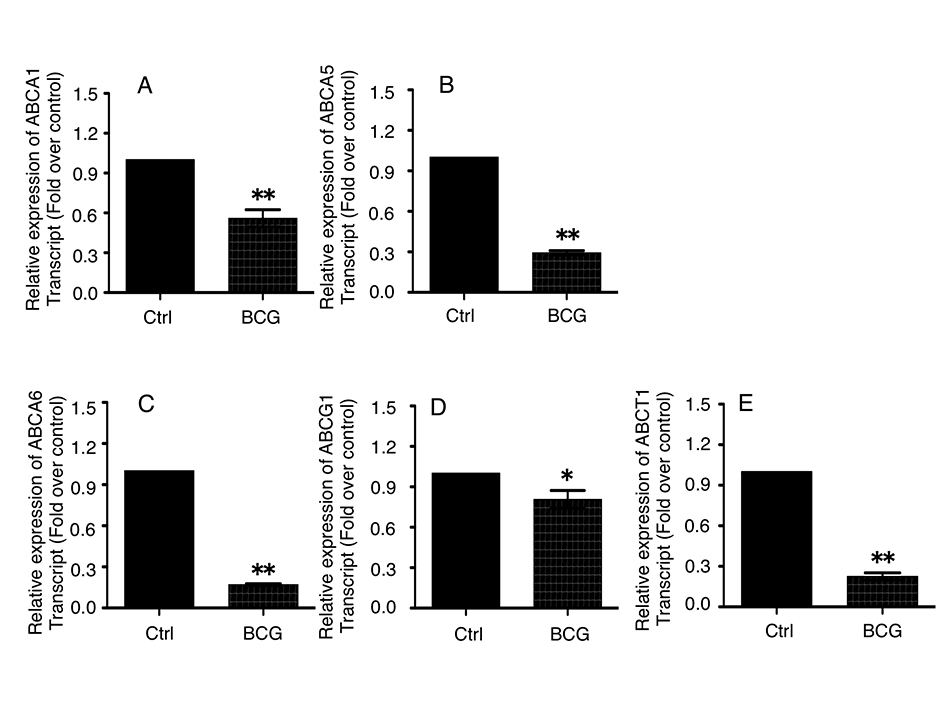
**

**Suppl. Figure S2. The BCG infection suppresses the expression of ABC-transporters and ACAT1 in bovine alveolar macrophages (AMs).** Bovine AMs were infected with BCG at MOI of 10 for 12 h, and the transcripts of ABC-transporters and ACAT1 was assessed by a qRT-PCR assay. (A-E) Inductions of indicated transcripts of bovine alveolar macrophages (AMs) infected with BCG. (A) Fold of changes of ABCA1 transcript over the non-infected cells; (B) Fold of changes of ABCA5 transcript over the non-infected cells; (C) Fold of changes of ABCA6 transcript over the non-infected cells; (D) Fold of changes of ABCG1 transcript over the non-infected cells; (E) Fold of changes of ACAT1 transcript over the non-infected cells. Data represent the mean ± the standard error of the mean (SEM) from three independent experiments. Compared to non-infection control, *: *p*<0.05, **: *p*<0.01.

Suppl. Table 1. Primer sets of qRT-PCR used in this study

| Genes | Gene ID | Sequence (5'-3') | Annealing Tm | Amplicon (bp) |
| --- | --- | --- | --- | --- |
| β-actin | 280979 | F: TGTGCTGTGGGTTTTTCTCC | 61°C | 138 |
|  |  | R: TCATCCTCATTGTTGGGTGG |  |  |
| ABCA5 | 510497 | F: TGTGCTGTGGGTTTTTCTCC | 62°C | 163 |
|  |  | R: TCATCCTCATTGTTGGGTGG |  |  |
| ABCA6 | 537351 | F: GGGCATTTTTTCTCTCGTTATC | 60°C | 94 |
|  |  | R: GAAGTGCTTGAGTTTGCTGGT |  |  |
| ABCA1 | 535379 | F: CACTGCCTCGCTAGCTTTAATT | 59°C | 172 |
|  |  | R: CATCTTGGCCATCATACTGTTT |  |  |
| ABCG1 | 510745 | F: AACCAACCATGCGCACTAAG | 61°C | 108 |
|  |  | R: AGAAGCACGGGGGAAATAAA |  |  |
| ACAT1 | 511082 | F: AATGAAGCCTTTAGTGTGGTCG | 60°C | 143 |
|  |  | R: TGAGCCAAATGGACGACAAT |  |  |

| Antigen | MW | Vendor | Dilution | Cat. # | Host |
| --- | --- | --- | --- | --- | --- |
| ATG5 | 35KDa | Proteintech | 1:500 | 10181-2-AP | Rabbit |
| ATG7 | 70KDa | Proteintech | 1:500 | 10088-2-AP | Rabbit |
| LC3 | 18KDa、14KDa | Proteintech | 1:500 | 14600-1-AP | Rabbit |
| Beclin1 | 60KDa | Proteintech | 1:500 | 11306-1-AP | Rabbit |
|  |  | CST | 1:800 | #3738 | Rabbit |
| ACAT1 | 42KDa | Proteintech | 1:500 | 16215-1-AP | Rabbit |
| ABCA1 | 254KDa | abcam | 1:1000 | ab18180 | Mouse |
| ABCA5 | 186KDa | Invitrogen | 1:1000 | PA5-42402 | Rabbit |
| ABCA6 | 183KDa | Invitrogen | 1:1000 | PA5-66902 | Rabbit |
| ABCG1 | 60KDa | Proteintech | 1:500 | 13578-1-AP | Rabbit |
| β-actin | 42KDa | Proteintech | 1:1000 | 20536-1-AP | Rabbit |

**Suppl. Table 2.** The information of used antibodies
